# Shiga Toxin 1 Induces on Lipopolysaccharide-Treated Astrocytes the Release of Tumor Necrosis Factor-alpha that Alter Brain-Like Endothelium Integrity

**DOI:** 10.1371/journal.ppat.1002632

**Published:** 2012-03-29

**Authors:** Verónica I. Landoni, Pablo Schierloh, Marcelo de Campos Nebel, Gabriela C. Fernández, Cecilia Calatayud, María J. Lapponi, Martín A. Isturiz

**Affiliations:** 1 Departamento de Inmunología, CONICET, Ciudad Autónoma de Buenos Aires, Argentina; 2 Departamento de Genética, CONICET, Ciudad Autónoma de Buenos Aires, Argentina; 3 Química Biológica, Facultad de Farmacia y Bioquímica, Universidad de Buenos Aires, Ciudad Autónoma de Buenos Aires, Argentina; 4 Laboratorio de Trombosis Experimental of Instituto de Medicina Experimental (IMEX), CONICET, Ciudad Autónoma de Buenos Aires, Argentina; University of California Los Angeles, United States of America

## Abstract

The hemolytic uremic syndrome (HUS) is characterized by hemolytic anemia, thrombocytopenia and renal dysfunction. The typical form of HUS is generally associated with infections by Gram-negative Shiga toxin (Stx)-producing Escherichia coli (STEC). Endothelial dysfunction induced by Stx is central, but bacterial lipopolysaccharide (LPS) and neutrophils (PMN) contribute to the pathophysiology. Although renal failure is characteristic of this syndrome, neurological complications occur in severe cases and is usually associated with death. Impaired blood-brain barrier (BBB) is associated with damage to cerebral endothelial cells (ECs) that comprise the BBB. Astrocytes (ASTs) are inflammatory cells in the brain and determine the BBB function. ASTs are in close proximity to ECs, hence the study of the effects of Stx1 and LPS on ASTs, and the influence of their response on ECs is essential. We have previously demonstrated that Stx1 and LPS induced activation of rat ASTs and the release of inflammatory factors such as TNF-α, nitric oxide and chemokines. Here, we demonstrate that rat ASTs-derived factors alter permeability of ECs with brain properties (HUVECd); suggesting that functional properties of BBB could also be affected. Additionally, these factors activate HUVECd and render them into a proagregant state promoting PMN and platelets adhesion. Moreover, these effects were dependent on ASTs secreted-TNF-α. Stx1 and LPS-induced ASTs response could influence brain ECs integrity and BBB function once Stx and factors associated to the STEC infection reach the brain parenchyma and therefore contribute to the development of the neuropathology observed in HUS.

## Author Summary

Hemolytic-uremic syndrome (HUS) is generally caused by Shiga toxin (Stx)-producing Escherichia coli but bacterial lipopolysaccharide (LPS) and neutrophils (PMN) contribute to the pathophysiology. Acute renal failure is the main feature of HUS, but in severe cases, patients develop neurological complications, which are usually associated with death. Although the mechanisms of neurological damage remain uncertain, alterations/injury of brain endothelial cells (ECs) which constitute the blood-brain barrier (BBB) is clear. Astrocytes (ASTs) are inflammatory cells enclosing ECs and are responsible of the normal function of the barrier. We have recently demonstrated that Stx1, one of the most common types of Stx, induce an inflammatory response in LPS-treated ASTs. We then study the effects of factors released by ASTs in response to LPS and/or Stx1 on brain-like ECs. We demonstrate that Stx1 induces in LPS-treated ASTs the release of factors that alter brain properties in ECs, including the permeability; turning them more susceptible to Stx1 toxic effects. Furthermore, they activate ECs, neutrophils (PMN) and platelets and render ECs into a proagregant state promoting PMN and platelet adhesion. Our results suggest that ASTs could influence brain ECs integrity and BBB function once Stx in combination with bacterial factors reach the brain parenchyma.

## Introduction

The epidemic form of hemolytic uremic syndrome (HUS), has been associated with enterohemorrhagic infections caused by Shiga toxin (Stx)-producing *Escherichia coli* (STEC) [1]. HUS is the most common cause of acute renal failure in children and is related to endothelial damage of kidney glomeruli and arterioles and epithelial cell damage induced by Stx, through the interaction with its globotriaosylceramide (Gb_3_) receptor [2]. Although Stx is the main pathogenic factor for HUS development, the inflammatory response is able to potentiate Stx toxicity. In fact, both bacterial lipopolysaccharide (LPS), and polymorphonuclear neutrophils (PMN) play an important role in the full development of HUS [3].

In severe cases of HUS, endothelial cell (ECs) damage is not limited to the kidney but extends to other organs, such as the brain. Central nervous system (CNS) complications are observed in about 30% of infant population with HUS and brain damage is the most common cause of death in this disease [4].

Brain ECs are part of the blood brain barrier (BBB), they restrict the entry of potentially harmful substances and leukocytes from the bloodstream. In fact, brain ECs damage is thought to be involved in the disruption of the BBB integrity observed in HUS. However, the pathogenesis of CNS impairment is not yet fully understood. Although human brain ECs are relative resistant to Stx effects in vitro, inflammatory stimuli markedly increase their sensitivity towards Stx toxicity by increasing Gb_3_ expression on these cells [5].

ASTs are inflammatory cells found throughout the CNS and are surrounding almost entirely the brain endothelium by terminal processes [6]. The interaction of ASTs with brain ECs determines the BBB function [7], as soluble factors released by ASTs can mediate not only the induction but also the maintenance of BBB properties in brain ECs [8], [9]. In response to brain injury, ASTs become activated and release inflammatory mediators altering the integrity and permeability of the BBB which can affect neuronal survival and tissue integrity [10], [11]. In addition, ASTs derived cytokines and chemokines can stimulate the peripheral immune system and attract peripheral inflammatory leukocytes to the site of injury [12]. We have recently demonstrated that Stx1 exerts a direct effect on ASTs although sensitization with LPS potentiates the effects induced by Stx1. Stx1 induces activation of ASTs and development of an inflammatory response characterized by the secretion of NO, TNF-α and chemokines that promote PMN attraction [13].

Given the critical anatomical disposition of ASTs and the influence they exert over ECs from BBB, we hypothesized that the effects induced by LPS and Stx1 on ASTs may contribute to the brain ECs damage observed in severe cases of HUS. Thus, the aim of this study was to evaluate the effects of soluble factors released by ASTs treated with Stx1 alone, or in combination with LPS on ECs with brain endothelium properties.

Here we demonstrate that rat ASTs treated with LPS and Stx1 release factors that affect the permeability of brain-like ECs and increase their susceptibility towards Stx1 cytotoxicity. Additionally, these factors activate ECs, PMN and platelets inducing a proinflammatory and prothrombic state that promotes PMN and platelet adhesion to ECs.

Together, our results suggest that ASTs could influence brain ECs integrity and BBB function once Stx in combination with bacterial factors reach the brain parenchyma.

## Results

### Conditioned media from ASTs induce on HUVEC properties of brain endothelium

In order to accurately model the human BBB, purified ECs obtained from human umbilical cord veins (HUVEC) were differentiated into ECs with properties of cerebral endothelium (HUVECd) by incubating them with conditioned media from ASTs (CM-ASTs). We assessed the induction of several properties that characterize brain ECs after 24 h incubation of HUVEC with CM-ASTs.

**Figure 1:**
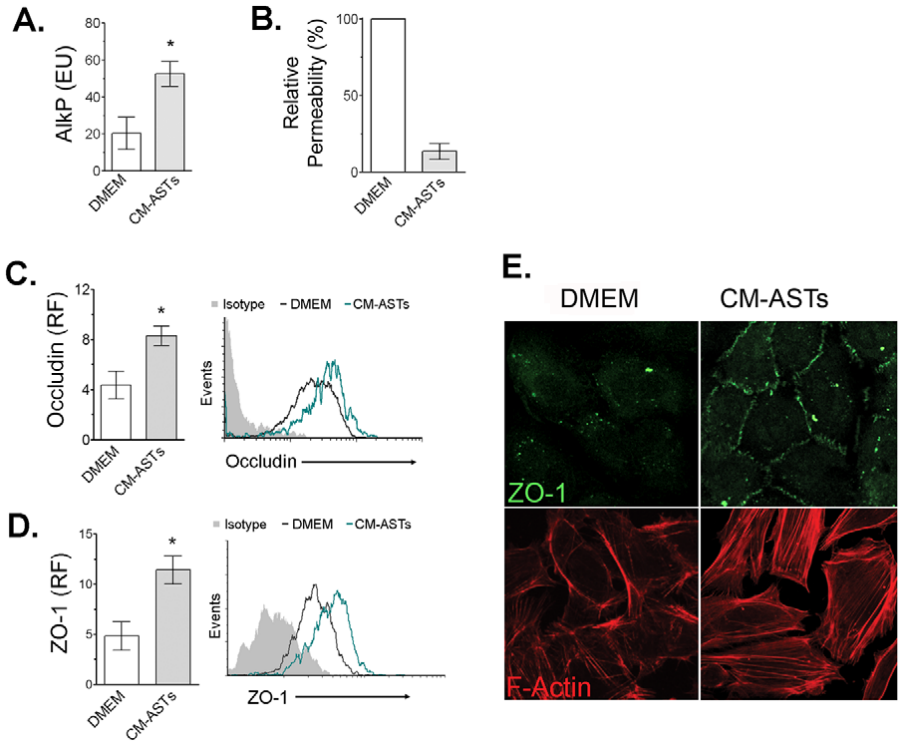
Astrocyte conditioned media (CM–ASTs) induce brain endothelium properties on HUVEC. HUVEC were culture with CM-ASTs (HUVECd) or complete medium DMEM (HUVEC) for 24 h. Thereafter, alkaline phosphatase (AlkP) activity (**A**) or transendothelial monolayer passage of horse radish peroxidase (HRP) (**B**) were spectrophotometrically assessed. Occludin (**C**) and ZO-1 (**D**) expression was FACS analyzed and cellular expression of ZO-1 and distribution of F-Actin were evaluated by microscopy (**E**) as described in M&M. **A**: Enzymatic units (EU) of AlkP specific activity (Mean ± SEM). * p<0.05 (CM-ASTs vs. DMEM, n = 4). **B**: % Relative permeability (Mean ± SEM, n = 3) of endothelial monolayer to HRP. **C and D**: Occludin and ZO-1 relative fluorescence (RF, median fluorescence intensity (MFI)/isotype control MFI) (Mean ± SEM).*p<0.05 (CM-ASTs vs. DMEM, n = 3). **E**: Photomicrography showing ZO-1 expression pattern of HUVEC (upper left panel) and HUVECd (upper right panel) of one representative experiment (n = 3). Photomicrography showing maximum intensity Z-projection of F-Actin distribution on HUVEC (lower Left panel) and HUVECd (lower right panel) of one representative experiment (n = 4).

Figure 1A shows a significant increase in the activity of alkaline phosphatase (AlkP) in HUVECd in comparison to HUVEC maintained in control medium (DMEM). The activity of this enzyme remained high during 72 hours, even though the CM-ASTs was removed after 24 hours of incubation (data not shown).

Moreover, as shown in Figure 1B, the permeability to the passage of horse radish peroxidase (HRP) was decreased in HUVECd.

Similarly, basal expression of tight junction (TJs) and TJs-associated proteins, occludin and ZO-1 respectively, were also increased in HUVECd (Figure 1C and 1D). Additionally, photomicrographs depicted in Figure 1E corroborated these results, where an intense expression of ZO-1 and its typical localization at junctional regions of the cell surface were only evident in HUVECd.

Finally, as ZO-1 is associated with actin filaments, we investigated the pattern of distribution of F-actin on HUVECd. Figure 1E shows structural changes in F-actin on cells cultured with CM-ASTs. While HUVEC maintained in DMEM showed an intense pattern of F-actin at the cell periphery (“belt” like distribution), the arrangement of F-actin in HUVECd is mainly organized in transcytoplasmatic parallel bundles of intense stress fibers, with a lower degree of peripheral distribution as expected for brain ECs.

Together, these results support the fact that soluble factors released by ASTs under basal conditions are able to transdifferentiate non-neural ECs into ECs with brain properties that simulates the endothelium from BBB.

### Stx1 induces on LPS sensitized-ASTs the release of toxic factors for HUVECd

ASTs response to Stx1 entering the brain parenchyma may include the secretion of toxic factors for brain ECs located in close proximity. In order to test this hypothesis, we determined the toxicity on HUVECd induced by untreated CM-ASTs (CM-Control) or CM-ASTs treated with LPS and/or Stx1 (CM-LPS, CM−Stx1 and CM−LPS+Stx1).

**Figure 2:**
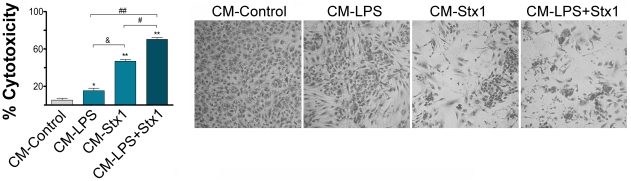
Increased HUVECd toxicity induced by factors released by LPS and/or Stx1-treated ASTs. HUVECd were stimulated for 72 h with CM-Control, CM-LPS, CM-Stx1 or CM-LPS+Stx1. Detached dead cells were washed away and the remaining cells were stained and counted by optical microscopy for toxicity evaluation. % of cytotoxicity respect unstimulated HUVECd (Mean ± SEM; n = 5), *p<0.01 (CM-LPS or CM-Stx1 or CM-LPS+Stx1 vs. CM-Control); & p<0.01 (CM-LPS or CM-LPS+Stx1 vs. CM-Stx1); ## p<0.01 (CM-LPS+Stx1 vs. CM-LPS). Representative microphotography of one independent experiment showing HUVECd cultures stained with crystal violet (n = 5).

After 24 h HUVECd confluent cultures were observed by optical microscopy to register any change in the cellular appearance associated with toxicity (shape, refringency or cellular detachment). Although no apparent changes were observed during the first 48 h, signs of toxicity were clearly evident at 72 h. Figure 2 shows a slight but significant toxicity induced on HUVECd incubated with CM−LPS compared with CM-Control. CM−Stx1 induced even more toxicity on HUVECd. However, maximal toxicity was observed when HUVECd were incubated with CM−LPS+Stx1.

These results suggest that Stx1 induces in LPS-sensitized ASTs the release of toxic factors that contribute to a long-term damage of endothelial cells with cerebral properties.

### Stx1 induces on LPS-sensitized ASTs the release of factors that increase Gb_3_ expression and sensitivity of HUVECd to Stx1

**Figure 3:**
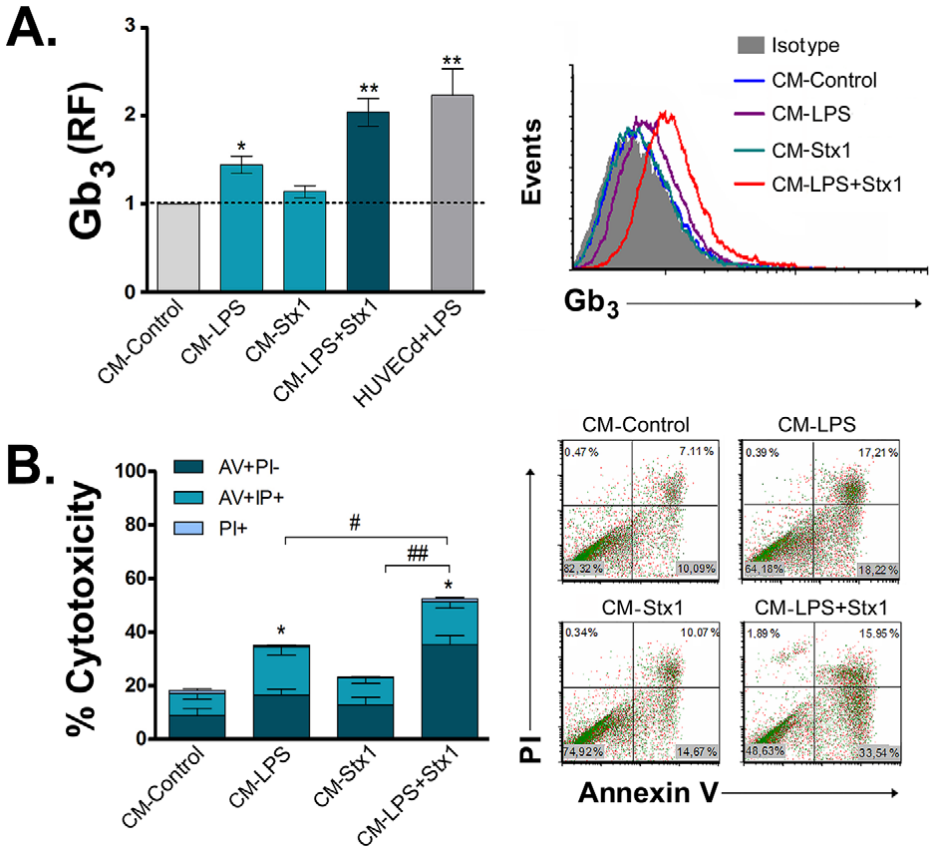
Factors released by Stx1 and/or LPS-treated ASTs increase Gb_3_ expression and Stx1 susceptibility in HUVECd. HUVECd were stimulated with CM-Control, CM−LPS, CM−Stx1, CM−LPS+Stx1 or with 0.5 µg/ml de LPS (HUVECd+LPS) for 24 h. Then stimulus was removed and Stx1 was added for another 18 h. Finally, Gb_3_ expression (**A**) and toxin induced cell death (**B**) were evaluated by flow cytometry (FACS). **A**: Relative Fluorescence (RF) of Gb_3_ (Mean fluorescence intensity relative to unstimulated HUVECd, Mean ± SEM). *p<0.05 (CM-Control vs. CM−LPS); **p<0.0002 (CM-Control vs. CM−LPS+Stx1 or HUVECd+LPS, n = 3). Left panel is a representative histogram plot of one independent experiment. **B**: % of total Stx1 induced cell death (Mean ± SEM) determined by PI/Annexin V binding assay. Total dead cells were defined as the sum of necrotic (PI+/AV−), late apoptotic (PI+/AV+) and early apoptotic (PI−/AV+) HUVECd cells. *p<0.05 (CM-Control vs. CM−LPS or CM−LPS+Stx1); ^#^p<0.05, ^##^p<0.001 (CM−LPS+Stx1 vs. CM−LPS and CM−LPS+Stx1 respectively, n = 4). The left panel represents a dot-plot of one independent experiment.

As inflammatory mediators can modulate the expression of the Stx receptor, the effect of released factors from ASTs treated with LPS and/or Stx1 on Gb_3_ expression was evaluated on HUVECd after a 24 h exposure. Figure 3A shows that the maximum increase of Gb_3_ was obtained when HUVECd were treated with CM−LPS+Stx1. As expected, HUVECd directly treated with LPS showed an up regulation of the Stx receptor.

To determine whether the enhanced Gb_3_ expression correlates with an increased toxicity induced by Stx1, HUVECd treated with the different CM-ASTs were exposed to a sublethal dose of Stx1. After 18 h, cell death was evaluated by Annexin V (AV) and propidium iodide (PI) staining (Figure 3B). In accordance to the results observed for the Gb_3_ expression, the percentage of total cell death (AV−PI+ plus AV+PI− plus AV+PI+) induced by Stx1 was increased in HUVECd exposed to CM−LPS. Moreover, the cultures treated with CM−LPS+Stx1 showed the highest susceptibility to Stx1. The analysis of the quadrants depicted in Figure 3B shows that the number of cells mapping in the necrotic quadrant (AV−/PI+) is relatively small and constant for all treatments, whereas most cell death is present in the early apoptotic (AV+/PI−) and late apoptotic (AV+/PI+) quadrants, indicating that apoptosis is the preferential mechanism accounting for HUVECd cell death in response to CM−LPS+Stx1.

Overall, these results indicate that Stx1 induces on LPS-sensitized ASTs the release of factors that sensitize HUVECd to the toxic effects of Stx1, and this correlates with an increased expression of Gb_3_ in HUVECd.

### Stx1 induces on LPS-sensitized ASTs the release of factors that alter the expression of tight junction proteins on HUVECd and increase toxin transendothelial translocation

**Figure 4:**
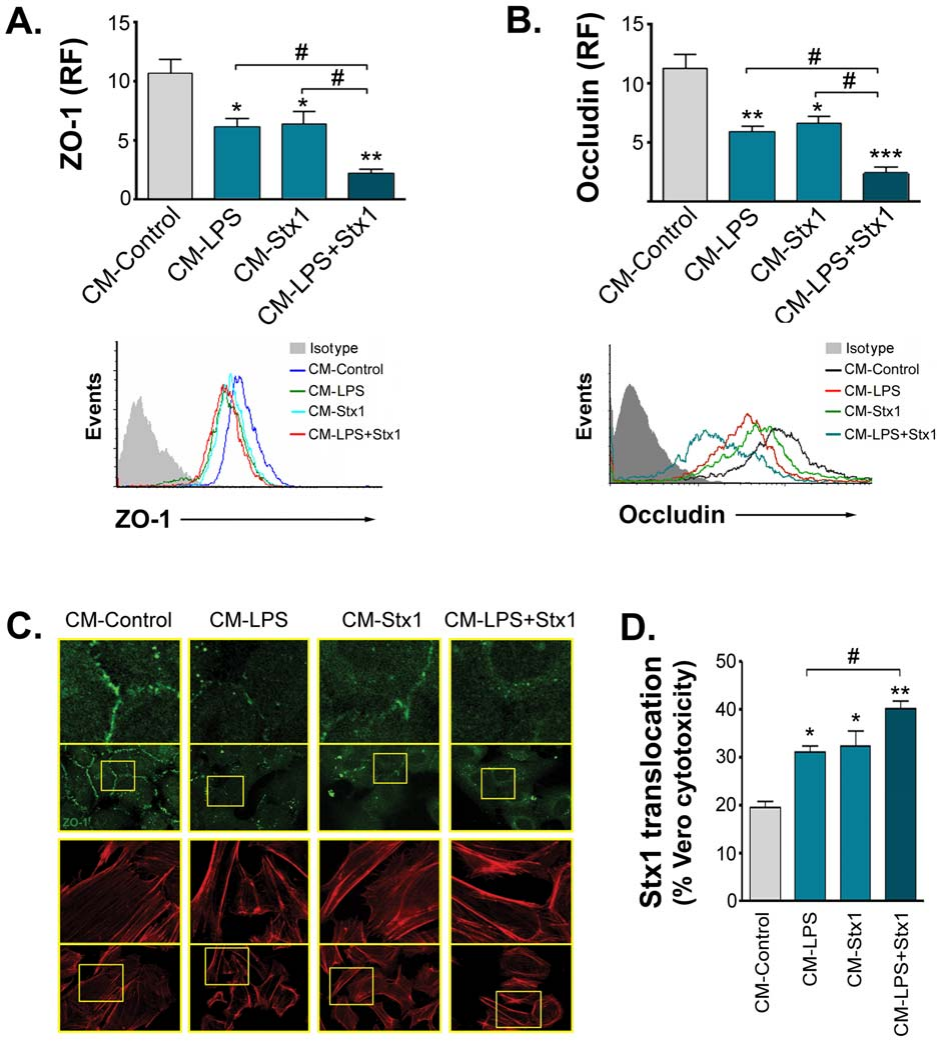
Factors released by LPS and/or Stx1-treated ASTs increase transendothelial permeability by reducing tight-junctions proteins expression on HUVECd. HUVECd seeded on culture plates (**A,B,C**) or in upper transwell chamber (**D**) were stimulated during 24 h with CM-Control, CM−LPS, CM−Stx1 or CM−LPS+Stx1. ZO-1 (**A**) and occludin (**B**) expression were evaluated by FACS. ZO-1 expression and F-Actin cellular distribution were evaluated by confocal microscopy (**C**). Stx1 passage across HUVECd monolayer was determined using the Vero cell cytotoxicity bioassay after 30 min of toxin translocation to the lower transwell chamber (**D**). (**A**) ZO-1 or (**B**) occludin relative median fluorescence intensity (RF) (Mean ± SEM, n = 4). *p<0.05 (CM−LPS or Stx1 vs. CM-Control); **p<0.001 (CM−LPS or CM−LPS+Stx1 vs. CM-Control); ***p<0.0001 (CM−LPS+Stx1 vs. CM-Control); ^##^p<0.001(CM−LPS+Stx1 vs. CM−LPS or CM−Stx1). Lower panel: a representative histogram of an independent experiment is shown. **C**: Microphotographs showing ZO-1 expression pattern on CM-treated HUVECd (upper panels) of an independent experiment (n = 3). Microphotographs showing maximum intensity Z-projection of F-Actin distribution on CM-treated HUVECd (lower panels) of an independent experiment (n = 4). **D**: Stx1 translocation measured as % Vero of cytotoxicity respect to Vero cell cultured in DMEM (Mean ± SEM; n = 3). *p<0.05 (CM-LPS or CM-Stx1 vs. CM-Control; **p<0.001 (CM−LPS+Stx1 vs. CM-Control); ^#^p<0.05 (CM−LPS+Stx1 vs. CM−LPS).

Several lines of evidence suggest that alterations in TJs, or proteins associated with them, could act as a possible mechanism leading to an increased BBB permeability [14]. Therefore, we evaluated the effect of CM-ASTs treated with LPS and/or Stx1 in the expression of proteins required for tight junction maintenance and BBB permeability. Figure 4A and 4B depicts that both CM−LPS and CM−Stx1 induced a significant decrease in the expression of ZO-1 and occludin on HUVECd compared to cells exposed to CM-Control. Nevertheless, this reduction was even more evident when HUVECd were stimulated with CM−LPS+Stx1. In agreement with these results, micrographs depicted in Figure 4C show the reduced ZO-1 expression. In this sense, the intense staining of ZO-1 observed in the periphery of adjacent HUVECd treated with CM-Control was significantly reduced in those cells exposed to CM-LPS or CM−Stx1, where only a few areas of low ZO-1 expression were evidenced. Furthermore, ZO-1 was absent in HUVECd stimulated with CM−LPS+Stx1, even at the contact areas between adjacent cells. In addition, micrographies shown in Figure 4C illustrate that F-actin in HUVECd exposed to CM-Control were more prominently arranged defining transcytoplasmatic stress fibers. On the contrary, in HUVECd stimulated with CM−LPS and/or Stx1, a peripheral localization of F-actin was observed. In addition, a cellular retraction was observed, with more pronounced intercellular spaces.

Factors released by ASTs may increase HUVECd permeability allowing the passage of Stx1 across the endothelium. To test this hypothesis we determined the translocation of Stx1 across HUVECd treated with the different conditioned media. HUVECd in transwell upper chambers were stimulated for 24 h with CM−LPS and/or Stx1. Then CM-ASTs were removed, cells were washed, and Stx1 was added. After 30 min. the medium in the lower chamber was recovered and the presence of Stx1 was determined by the Vero toxicity bioassay, a cell line highly sensitive to the toxin. Figure 4D shows a significant increase in the percentage of Vero cytotoxicity mediated by Stx1-translocated through HUVECd monolayer pretreated with CM−LPS or CM−Stx1. This increase was even higher for HUVECd incubated with CM−LPS+Stx1.

These results indicate that factors released by ASTs sensitized with LPS and treated with Stx1 altered the expression of TJs proteins and TJs-associated proteins on HUVECd, and induced an increase of HUVECd permeability, allowing the translocation of Stx1 across the endothelium.

### ASTs treated with LPS and Stx1 release factors that induce activation of HUVECd

In order to determine whether factors released by ASTs treated with LPS and/or Stx1 induce activation of HUVECd, we evaluated the expression of adhesion molecules such as ICAM-1 and E-selectin, and the release of procoagulant molecules, such as the von Willebrand Factor (vWF).

**Figure 5:**
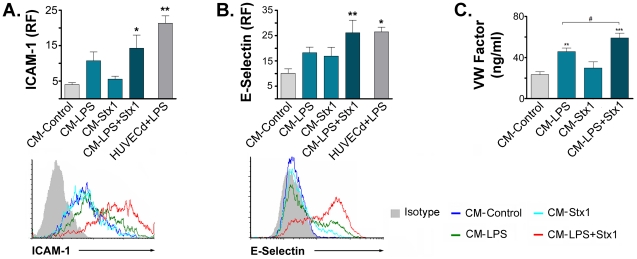
Factors released by LPS and Stx1-treated ASTs increased HUVECd activation. HUVECd were stimulated with CM-Control, CM−LPS, CM−Stx1, CM−LPS+Stx1 or with 0.5 µg/ml de LPS (HUVECd+LPS). ICAM-1 (**A**) and E-selectin (**B**) expression was evaluated by FACS after 12 h or 3 h of stimulation, respectively. The release of von Willebrand Factor (vWF) was quantified by ELISA (**C**). **A**: ICAM-1 relative median fluorescence (RF) (Mean ± SEM, n = 4). *p<0.05 (LPS+Stx1 vs. Control); **p<0.001 (HUVEC+LPS vs. CM-Control, n = 3). **B**: E-selectin relative median fluorescence (RF) (Mean ± SEM, n = 4). **p<0.005 (CM−LPS+Stx1 vs. Control); *p<0.05 (CM−HUVEC+LPS vs. CM-Control, n = 4). Representative histograms of one independent experiment were plotted in the lower panels. **C**: Concentration (ng/ml) of vWF released (Mean ± SEM, n = 6). **p<0.01 (CM−LPS vs. CM-Control); **p<0.001 (CM−LPS+Stx1 vs. CM-Control); ^#^p<0.05 (CM−LPS+Stx1 vs. CM−LPS).

Figures 5A and B show that the expression of ICAM-1 and E-selectin was significantly increased exclusively on HUVECd stimulated with CM−LPS+Stx1. The expression of these molecules was similar to that obtained for HUVECd stimulated directly with LPS. Figure 5C shows an increase in vWF secretion induced by CM−LPS. Nevertheless, the highest secretion was observed when HUVECd were stimulated with CM−LPS+Stx1.

These results suggest that Stx1 causes the release of factors on LPS-sensitized ASTs that induce the activation and a prothrombotic state on HUVECd.

### ASTs treated with LPS and Stx1 release factors that induce activation of PMN and increase PMN transendothelial migration

**Figure 6:**
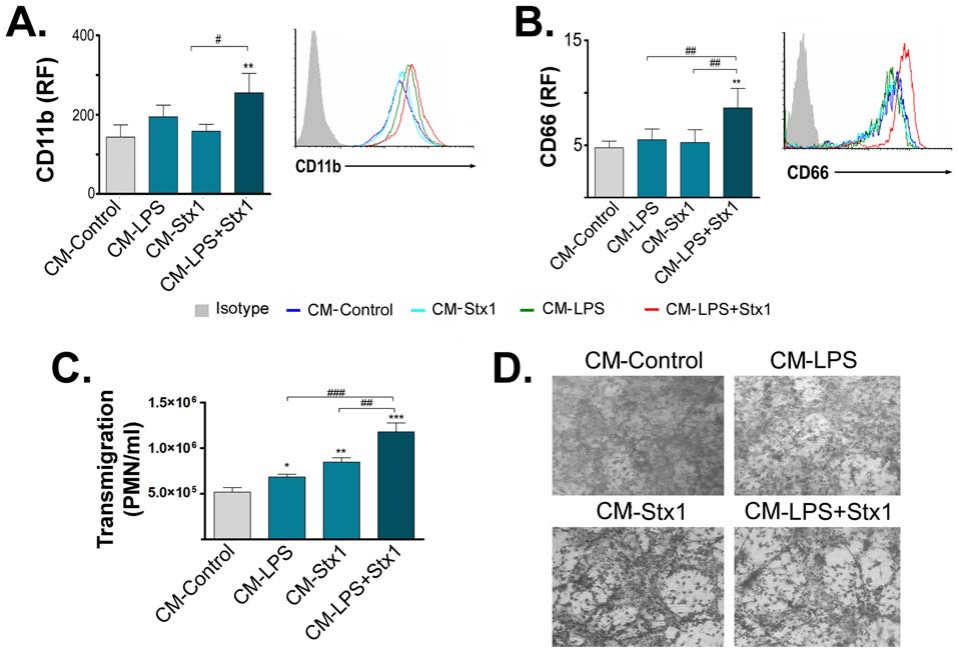
Factors released by LPS and Stx1-treated ASTs induce activation and migration of PMN through HUVECd. PMN were stimulated with CM-Control, CM−LPS, CM−Stx1 or CM−LPS+Stx1. CD11b (**A**) or CD66b (**B**) expressions were evaluated by FACS at 40 or 90 min after stimulation, respectively. In (**C**), HUVECd seeded on the upper chamber of a transwell were stimulated with CM-Control, CM−LPS, CM−Stx1 or CM−LPS+Stx1 for 24 h. PMN migration across the HUVECd monolayer was determined microscopically after 1.5 h of co-culture. Disruption of HUVECd monolayer integrity was evaluated after PMN transmigration by staining HUVECd with May Grünwald-Giemsa (**D**). **A**: CD11b relative median fluorescence (RF) (Mean ± SEM, n = 4). **p<0.005 (CM−LPS+Stx1 vs. CM-Control); ^#^p<0.05 (CM−LPS+Stx1 vs. CM−Stx1). Representative histogram of one independent experiment is plotted in the right panel. **B**: CD66b relative median fluorescence (RF) (Mean ± SEM, n = 4). **p<0.001 (LPS+Stx1 vs. Control); ^##^p<0.005 (CM−LPS+Stx1 vs. CM−Stx1). A Representative histogram of one independent experiment is plotted in the right panel. **C**: Transmigration of PMN (PMN/ml) (Mean ± SEM, n = 3). *p<0.05 (CM−LPS vs. CM-Control); **p<0.01 (CM−Stx1 or CM−LPS+Stx1 vs. CM-Control); ^##^p<0.01 (CM−LPS+Stx1 vs. CM−Stx1); ^###^p<0.001 (CM−LPS+Stx1 vs. CM−LPS, n = 3). **D**: Representative microphotographs depicting HUVECd monolayer disruption after PMN transmigration (n = 3).

Numerous studies have suggested that activation of leukocytes is critical for endothelial damage. Therefore, we analyzed whether factors released by ASTs treated with LPS and/or Stx1 induce PMN activation. Purified PMN were incubated with the different CM-ASTs and the expression of PMN-activation markers was evaluated. An increase in the expression of CD11b (Figure 6A) and CD66b (Figure 6B) was found on PMN stimulated with CM−LPS+Stx1 compared with CM-Control.

The production of cytokines and chemokines by brain ECs and ASTs can account for both recruitment and activation of leukocytes [15], [16]. Therefore, we assessed whether factors released from ASTs treated with LPS and/or Stx1 were able to promote PMN migration across HUVECd. For this purpose, HUVECd placed in transwell upper chambers were stimulated for 24 h with CM−LPS and/or Stx1. Afterward, PMN were added in the upper chamber and the number of migrated PMN in the lower chamber was determined after 1.5 h. Results depicted in Figure 6C reveal that the concentration of PMN that migrated through the endothelial monolayer increased significantly with the CM−LPS or CM−Stx1 compared with CM-Control. However, CM−LPS+Stx1 induced the maximal PMN transmigration.

As the passage of leukocytes through ECs may contribute to the disruption of the cellular monolayer, HUVECd were vigorously washed in order to eliminate non-migrated PMN and then monolayers were stained. Micrographies depicted in Figure 6D show that the monolayer architecture of HUVECd was disrupted by transmigration of PMN on those cultures treated with CM−LPS or CM−Stx1. However, disruption of HUVECd architecture as a result of PMN transmigration was higher when HUVECd were stimulated with CM−LPS+Stx1. These effects were not observed in HUVECd stimulated with the different CM-ASTs in the absence of PMN, dismissing the possibility that the CM-ASTs alone could cause these effects (data not shown).

In summary, these results suggest that Stx1 induces on LPS-sensitized ASTs the release of factors that activate PMN and promote their transmigration through endothelium, causing in turn, the disruption of the endothelial monolayer.

### Stx1 induces on ASTs sensitized with LPS the release of factors that increase the adhesion of PMN and sensitivity to PMN-mediated damage in HUVECd

PMN-mediated endothelial damage can seriously compromise vasculature and associated tissue functions. The adhesion of PMN to endothelium and the consequent cytotoxicity is magnified by the expression of endothelial ICAM-1 and E-selectin. Therefore, we tested whether ASTs exposed to the toxin could promote PMN adhesion to HUVECd and damage. HUVECd were stimulated with CM−LPS and/or Stx1. After removal of the stimulus, purified PMN were added and non-adhered PMN were removed 3 h later by vigorous washing. The percentage of PMN-derived alkaline phosphatase activity (AlkP) in the remaining attached cells was measured in order to evaluate PMN adhesion to HUVECd. On the other hand, to assess PMN-mediated cytotoxicity, the co-cultures were washed out after 8 h and stained with crystal violet. The percentage of cytotoxicity was determined microscopically by counting the remaining attached HUVECd. HUVECd were easily distinguishable from PMN because of their differences in shape and staining intensity.

**Figure 7:**
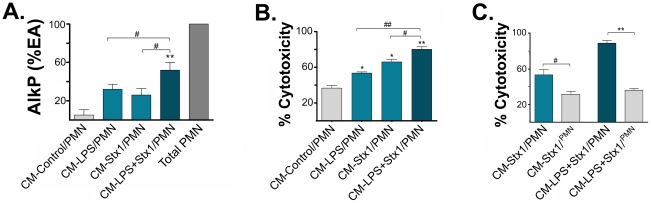
Factors released by LPS and/or Stx1-treated ASTs induce increased PMN adhesion and HUVECd toxicity. HUVECd were stimulated with CM-Control, CM−LPS, CM−Stx1 or CM−LPS+Stx1 for 24 h. Then, PMN were seeded onto pretreated HUVECd (CM-Control/PMN, CM−LPS/PMN; CM−Stx1/PMN or CM−LPS+Stx1/PMN). (**A**) After 3 h of co-culture, non-adhered PMN were washed away and the percentage of enzymatic activity (% EA) of alkaline phosphatase (AlkP) from adhered PMN was determined by spectrophotometry. As a control of maximal PMN adhesion, total PMN on untreated HUVECd were left unwashed (Total PMN). (**B**) To determine PMN mediated endothelial toxicity, PMN and dead HUVECd were washed after 8 h of co-culture and cultures were stained with crystal violet and the % of cytotoxicity was determined microscopically by counting the remaining attached HUVECd. In (**C**), contact dependency of PMN-mediated cytotoxicity was evaluated as (B), seeding PMN on the upper chamber of a transwell (“PMN” written in superscript) to avoid contact with CM-ASTs-stimulated HUVECd seeded in the lower chamber (CM−Stx1/^PMN^ or CM−LPS+Stx1/^PMN^). **A**: % of AlkP activity (% EA) respect to total PMN (Mean ± SEM, n = 6). **p<0.001 (CM−LPS+Stx1/PMN vs. CM-Control/PMN); #p<0.05 (CM−LPS+Stx1/PMN vs. CM−LPS o CM−Stx1/PMN). **B**: % of Cytotoxicity respect to HUVECd without PMN (Mean ± SEM, n = 3). *p<0.05 (LPS o Stx1 vs. Control); **p<0.001 (CM−LPS+Stx1/PMN vs. CM-Control/PMN); #p<0.01 (CM−LPS+Stx1/PMN vs. CM−Stx1/PMN); ##p<0.01 (CM−LPS+Stx1/PMN vs. CM−LPS/PMN). **C**: % of Cytotoxicity respect to HUVECd without PMN (Mean ± SEM, n = 2). **p<0.0001 (CM−LPS+Stx1/PMN vs. CM−LPS+Stx1/^PMN^); #p<0.01 (CM−Stx1/PMN vs. CM−Stx1/^PMN^).

Figure 7A shows an increase in the percentage of PMN-derived AlkP activity when HUVECd were stimulated with CM−LPS+Stx1. In addition, Figure 7B shows that the CM-LPS or CM−Stx1 sensitize HUVECd to PMN-mediated toxicity in comparison to HUVECd stimulated with CM-Control. However, this effect was further induced when HUVECd were stimulated with CM−LPS+Stx1. In order to determine whether PMN-mediated cytotoxicity is dependent on the direct interaction with HUVECd, we performed the same experiment but seeding PMN in the upper chamber of a transwell. Figure 7C shows that under this condition PMN-mediated cytotoxicity was avoided.

To sum up, these results indicate that Stx1 induces on LPS-sensitized ASTs the release of factors that promote PMN adhesion to HUVECd and increase their susceptibility to PMN-mediated damage, which depends on PMN contact with HUVECd.

### Stx1 induces on LPS-sensitized ASTs the release of factors that activate platelets and promote their adhesion to HUVECd

**Figure 8:**
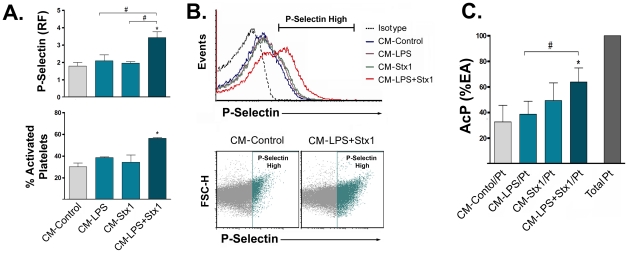
LPS and Stx1-treated ASTs release factors that increase platelet activation and adhesion to HUVECd. Platelets or HUVECd were stimulated with CM-Control, CM−LPS, CM−Stx1 or CM−LPS+Stx1. After 1.5 h density expression of P-selectin and the % of activated platelets was determined by FACS (**A** and **B**). To determine platelets adhesion to CM-stimulated HUVECd (**C**), platelets were seeded on stimulated HUVECd (CM-Control/Pt, CM−LPS/Pt, CM−Stx1/Pt, CM−LPS+Stx1/Pt). Free platelets were washed away after 1.5 h and the platelet acid phosphatase (AcP) percentage of enzymatic activity (% EA) was determined by spectrophotometry. As control of maximal platelet adhesion, total platelets on untreated HUVECd were left unwashed (Total Pt). **A**: P-selectin relative median fluorescence (RF) (Mean ± SEM, n = 6). *p<0.05 (CM−LPS+Stx1 vs. CM-Control); ^#^p<0.05 (CM−LPS+Stx1 vs. CM−Stx1 or CM−LPS) (Upper graph). % of activated platelets was defined by high P-selectin expressing subpopulation. *p<0.05 (CM−LPS+Stx1 vs. CM-Control) (Lower graph). **B**: Representative FACS analysis of an independent experiment showing histogram and scatter plots. **C**: AcP (% EA) respect to % EA of Total Pt (Mean ± SEM, n = 6). *p<0.05 (CM−LPS+Stx1/Pt vs. CM-Control/Pt); ^#^p<0.05 (CM−LPS+Stx1/Pt vs. CM−LPS/Pt, n = 6).

Under physiological conditions the endothelium produces many substances that prevent platelets activation and blood clots of fibrin [17]. In this sense, ASTs surrounding compromised endothelium could contribute to platelet activation/adhesion and subsequent brain microthrombi generation. In order to test this hypothesis, we determined whether factors released by ASTs treated with LPS and/or Stx1 induce platelets activation and adhesion to endothelium. As shown in Figure 8A expression of P-selectin and the percentage of activated platelets were increased by the CM−LPS+Stx1. To assess the platelets adhesion to endothelium, HUVECd were stimulated with CM−LPS and/or Stx1 for 24 h, and platelets were seeded on HUVECd. After 1.5 h, free platelets were removed by repeated washings, and the remaining adherent platelets were assessed by measuring acid phosphatase activity (AcP). The figure 8C shows an increase in the percentage of AcP activity induced by CM−LPS+Stx1.

Results indicate that in response to Stx1, LPS-sensitized ASTs released factors that activate platelets and promote their adhesion to HUVECd.

### The inhibition of NF-κB or the blockage of secreted TNF-α in ASTs exposed to LPS and Stx1 abrogated the response of HUVECd and platelets and PMN

**Figure 9:**
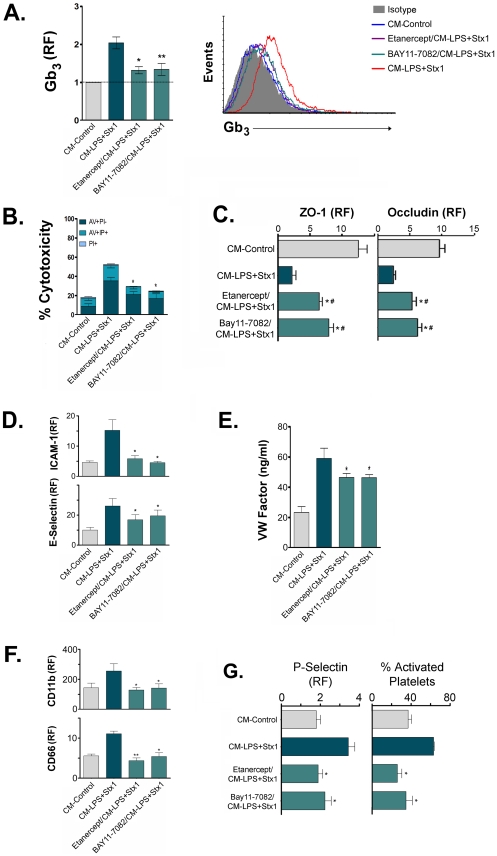
NF-κB inhibition or TNF-α blockade in LPS and Stx1-treated ASTs inhibit the effects of CM-ASTs. HUVECd, PMN or platelets were stimulated with CM-Control, or CM−LPS+Stx1 or CM of ASTs treated with BAY11-7082 o Etanercept before the addition of LPS and Stx1 (CM− LPS+Stx1/BAY 11-7082; CM-Etanercept/LPS+Stx1). **A**: HUVECd Gb_3_ relative median fluorescence (RF) (Mean ± SEM). *p<0.05 (CM−LPS+Stx1 vs. CM-Etanercept/LPS+Stx1); **p<0.01 (CM−LPS+Stx1 vs. CM-BAY 11-7082/LPS+Stx, n = 3). Histogram of an independent experiment is plotted in the right panel. **B**: HUVECd % Cytotoxicity (Mean ± SEM, n = 3) is determined by PI/Annexin V binding assay. *p<0.05 (CM−LPS+Stx1 vs. CM-Etanercept/LPS+Stx1); **p<0.01 (CM−LPS+Stx1 vs CM-BAY11-7082/LPS+Stx1). **C**: HUVECd ZO-1 or occludin relative median fluorescence (RF) (Mean ± SEM, n = 4). *p<0.05 (CM−LPS+Stx1 vs. CM-Etanercept or BAY11-7082/LPS+Stx1). **D**: HUVECd ICAM-1 or E-selectin relative median fluorescence (RF) (Mean ± SEM, n = 4). *p<0.05 (CM−LPS+Stx1 vs. CM-Etanercept or BAY11-7082/LPS+Stx1, n = 3). **E**: vWF release (ng/ml) (Mean ± SEM, n = 4). *p<0.05 (CM−LPS+Stx1 vs. CM-Etanercept or BAY11-7082/LPS+Stx1. **F**: PMN RF of CD11b or CD66b (Mean ± SEM, n = 4). *p<0.05 (CM−LPS+Stx1 vs. CM-Etanercept o BAY11-7082/LPS+Stx1); **p<0.01 (CM−LPS+Stx1 vs. CM-Etanercept/LPS+Stx1). **G**: Platelet RF of P-selectin and % of activated platelets (Mean ± SEM, n = 6). *p<0.05 (CM−LPS+Stx1 vs. CM-Etanercept or BAY11-7082/LPS+Stx1).

We have previously determined that inhibiting NF-kB with BAY 11-7082 or blocking secreted TNF-α activity with Etanercept prevented the activation and the inflammatory response on LPS-sensitized ASTs exposed to Stx1 [13]. Therefore, we investigated whether the effects observed in HUVECd, PMN and platelets were dependent on NF-κB activation, and particularly on the production of TNF-α by ASTs stimulated with LPS and Stx1. Thus, the experiments were conducted with CM-ASTs of ASTs that were pretreated with Etanercept or BAY 11-7082 before the addition of LPS and Stx1. As shown in Figure 9, under these conditions neither the increment in Gb_3_ expression (Figure 9A) nor the increased sensitivity to the toxin (Figure 9B) was observed in HUVECd. Likewise, the declined expression of ZO-1 or occludin were not detected (Figure 9C). A similar behavior was found when endothelial activation was analyzed through ICAM-1 and E-selectin expression (Figure 9D) and the release of vWF (Figure 9E). Furthermore, neither PMN (Figure 9F) nor platelet (Figure 9G) activation was observed.

Overall, the results indicate that NF-κB activation on LPS+Stx1-treated ASTs is necessary for the secretion of factors that induce activation of HUVECd, PMN and platelets, as well as for the increased expression of the Stx1 receptor and sensitivity to the toxin in HUVECd. Moreover, TNF-α seems to mediate these effects.

## Discussion

CNS complications are recognized as a major determinant of morbidity and mortality in the acute phase of STEC infections. Although, the pathogenesis of CNS involvement is not yet fully understood, the disruption and/or increased permeability of the BBB are central events in the CNS complications observed during the acute phase of HUS [18]. Stx is a macromolecule, and although in normal condition it should not be able to enter to brain parenchyma, studies in animal models have demonstrated that the toxin crosses the barrier. Moreover, the toxin have been found associated not only to brain ECs but also on parenchymal cells close to perivascular spaces including neurons and ASTs [19], [20], [21] suggesting that during STEC infection the BBB is altered. In addition, several in vivo studies found Stx in spinal cord fluid during the initials hours after toxin systemic inoculation, whereas pathological changes of blood vessels are noted at later stages [22]. In line with this, neuronal abnormalities appear before any vascular affection, suggesting the importance of events happening on the neuronal side on the outcome of the vascular pathology observed in HUS.

Even though neurological commitment is epidemiologically related to Stx2 variant [23], growing experimental evidence demonstrated the neurotoxicity of Stx1 [13], [24], [25], [26], [27], [28], [29]. Moreover, Stx1 have been shown to be in some models, even more neurotoxic than Stx2 [30].

Astrocytes (ASTs) are the most abundant inflammatory cells [31], [32], they are surrounding the cerebral endothelium and their interaction with ECs determines the BBB phenotype and function [8], [9], [33], [34]. ASTs are therefore in a critical position to influence brain ECs integrity and the BBB function, once Stx and factors associated to the STEC infection reach the brain parenchyma. Although the astrocytic inflammatory response elicited by Stx *in vivo* or in HUS patients has not been studied until the moment, there are two reports in the literature using animal models that demonstrated ASTs activation and alteration after Stx inoculation [33], [34]. In addition, local production of TNF-α has been described in mouse brains after STEC infection [35]. We have recently demonstrate that Stx1 exerts a direct action on rat ASTs, although sensitization with LPS potentiates Stx1-induced effects, by means of increasing Gb3 expression, revealing activation of ASTs and the development of an inflammatory response characterized by the secretion of nitric oxide (NO), TNF-α and chemokines that promote PMN attraction. Moreover, ASTs derived TNF-α is a pivotal effector molecule that amplifies the Stx1 effects on LPS-sensitized ASTs [13]. Therefore, it is highly probable that mediators released by activated AST in response to Stx (and LPS) are influencing ECs functionality *in vivo*.

In the present work we seek to obtain new knowledge for the role of ASTs on brain endothelial dysfunction in an attempt to further address the contribution of the local elicited inflammatory response to the neurophatology of HUS.

Our model uses conditioned-media (CM) from rat ASTs (that express the Stx receptor Gb_3_), whereas ASTs from human biopsies were found negative for Gb_3_ expression [36]. However, activated human ASTs/astrocytoma cells do express Gb_3_ [37]. On the other hand, the LPS receptor (TLR4) has been shown to be expressed in human ASTs [38], [39], [40]. Therefore, although experiments using human primary ASTs are necessary to confirm our current results, we can speculate that during the disease process activation of ASTs by LPS, systemic inflammatory mediators, or other bacterial products, may lead to the induction of Gb_3_ in ASTs, triggering a local inflammatory response that will be, in turn, amplified by Stx. In this context, the results originated from the treatments with CM−LPS and CM−LPS+Stx1 may most likely represent the HUS scenario.

Peripheral endothelial cells can be induced to differentiate into brain capillary ECs by soluble factors released by ASTs [7], [36], [37], [38], [39]. Here, we differentiated HUVEC into ECs that adopt characteristics that coincide with those present in human brain ECs (HUVECd), which allowed us modeling more accurately human BBB. In this regard, we observed an increase in AlkP activity, an increased expression of ZO-1 and occludin and a reorganization of F-actin. These observations are consistent with those reported by others [38], [39]. In addition, we observed a decreased permeability to horse radish peroxidase (HRP) in HUVECd when compare to non-differentiated HUVEC, similar to the decrease in permeability observed in a comparative study using brain microvascular ECs and HUVEC [40]. Although we are aware of the limitations of our model, since HUVECd are not exactly ECs from brain origin, the use of human primary brain cells is restricted by the unavailability of experimental material, which is usually obtained from surgical material and often cannot be considered as “healthy” tissue [41], [42]. Additionally, some characteristics of the in vivo BBB are lost in culture and this is compensated by co-culturing with ASTs or their CM [8], [43]. On the other hand, the use of immortalized brain endothelial cell lines does not assure an exactly similar behavior in culture as in the brain. An advantage of using HUVECd primary cultures obtained from HUVECs of different donors is that the heterogeneity of responses that exist among different individuals is maintained, and the variability obtained in the results better represents the variability found in HUS patients, in contrast of using a cell line with the same genetic background. Taken all this considerations into account, we consider that brain properties elicited in HUVECd, by incubation of HUVEC with CM of ASTs, are enough representative of brain ECs.

Then, in order to evaluate if the astrocytic response to LPS and/or Stx1 could altered properties of ECs forming the BBB, we studied the effects of soluble factors released by ASTs exposed to LPS and/or Stx1 on HUVECd. We found that TNF-α released from LPS-sensitized ASTs treated with Stx1 was able to increase Gb_3_ expression on HUVECd and this correlated with a higher susceptibility towards Stx1 toxic effects. These findings are in agreement with other reports using human brain ECs [27], [44].

On the other hand, LPS-sensitized ASTs stimulated with Stx1 released factors that, only in the long term, turn out to be toxic for HUVECd. Although astrocytic factors responsible for HUVECd's toxicity were not determined in this work, TNF-α has been proposed as the molecule responsible for ASTs mediated cytotoxicity on oligodendrocyte [45] and neurons [46]. However, *in vitro*, TNF-α is not toxic for brain ECs cultures by its own, but it resulted in a surprising synergism when combined with reactive species [47]. In this respect, NO liberated in response to LPS and Stx1 may act in combination with TNF-α inducing long term HUVECd death. Another plausible explanation is that the protein synthesis inhibiting activity of Stx1 prevents the expression of a host response factor by ASTs that is necessary for maintenance of EC viability [48], [49], [50]. In this sense, down-regulation of cell survival factors, in addition to the release of cytotoxic factors, may also contribute to long term ECs death.

The commitment of the TJs is a distinctive characteristic in neuroinflamatories diseases [51]. Numerous inflammatory substances modulate the permeability of the BBB, including TNF-α, NO and LPS [52], [53]. *In vivo* studies have demonstrated that an increment in the barrier permeability is associated with low levels of expression of ZO-1, occludin and actin filaments [54]. Here we determined that factors released by ASTs in response to LPS or Stx1, and especially by the combination of LPS and Stx1, induced an important decrease in occludin and ZO-1 expression in HUVECd. Moreover, this correlated with a significant peripheral localization of the ZO-1 protein, which has been shown to impact on the function of the BBB. Even though there are no concrete evidences that determine how this redistribution happens, it is believed to be associated with the reorganization of actin filaments, which are connected to the proteins of the TJs complex through ZO-1 [55], [56]. The results shown in this work indicate, for the first time, that ASTs inflammatory response induced by LPS and Stx and particularly ASTs-derived TNF-α, alters these molecules contributing to the increased endothelial permeability observed in HUS. Although *in vivo* studies must be performed to corroborate this hypothesis, studies on other cerebral pathologies support this possibility [53], [57]. In this sense, Álvarez *et al.* demonstrated in a murine model of cerebral inflammation that activated ASTs expressing BBB regulatory cytokines were juxtaposed to blood vessels exhibiting increased permeability [58].

An additional factor that can contribute to the loss of integrity of the BBB is the migration of PMN induced by chemotactic stimuli [59]. Migration across the BBB of PMN may initiate pathogenic events, leading to microvascular plugging, stasis, and thrombosis [60], [61]. PMN may migrate across the endothelial monolayer at cell-cell junctions by a paracellular route and/or by a transcellular pathway that involves migration of PMN at non-junctional locations. Here, we found an augmented migration of PMN across HUVECd treated with CM−LPS+Stx1. Given the increased permeability observed in HUVECd treated with CM−LPS+Stx1, and in concordance with a markedly reduction of tight junction proteins (ZO-1 and occludin), we can speculate that PMN more likely take a paracellular route to transmigrate, since this route is highly dependent on barrier function deregulation. Moreover, it has been recently demonstrated that PMN preferentially migrate across the BBB via the transcellular route when the barrier function is intact [62] Although PMN transmigration is not usually associated with disruption of the endothelial linearity, the presence of additional factors that induce PMN degranulation, such as TNF-α, can cause destruction of the vascular endothelial architecture due to the release of proteolytic enzymes [63], [64]. The results obtained here indicate that, in addition to the increased endothelial permeability, PMN transmigration induced by factors secreted by ASTs in response to LPS and Stx1 was associated to the destruction of the endothelial monolayer linearity, compromising even more the integrity of the BBB. Additionally, factors released by treated ASTs induced PMN-mediated endothelial toxicity, and in agreement with other reports [65], [66], this was dependent on the close interaction/contact between HUVECd and PMN.

All together, these results indicate that the systemic and local (from perivascular ASTs) inflammatory responses increase the BBB permeability, and at the same time, potentiate the damage of ECs triggered by Stx and factors associated to the infection.

Endothelial dysfunction is crucial for the development of microangiopathic injuries in HUS [67], [68], and a vast bibliography suggests that the interaction between activated leukocytes (especially PMN), platelets and ECs amplifies and extends renal damage [66], [69], [70]. However, so far no work regarding HUS-associated neuropathology contemplates the contribution of the intracerebral inflammation and its effect on ECs that comprise the BBB. Among the critical events commonly involved in CNS affection in HUS, edema, microthrombi and ischemic changes are found [71], [72]. Autopsy material from patients with HUS reveals thrombosis of capillaries in kidney, lung, liver and brain. Both damage and stimulation with inflammatory mediators suppress the anticoagulant properties of endothelium, promoting a procoagulant condition. We determined that factors secreted by ASTs treated with LPS and Stx1 directly induced HUVECd, PMN and platelet activation, and increased both PMN and platelet adhesion to activated HUVECd. Therefore, these events may contribute to the cerebral inflammation in HUS triggering the formation of thrombi and altering ECs integrity. Moreover, activation of HUVECd, PMN and platelets was not observed when ASTs were pretreated with BAY 11-7082 and exposed to LPS and Stx1, suggesting that inflammatory factors secreted by means of NF-kB activation were responsible for the effects observed in these cells. Furthermore, the blockade of ASTs released TNF-α by Etanercept also prevented these effects.

The results presented herein suggest that NF-κB and TNF-α could be target molecules to prevent or diminish the CNS complication observed in HUS patients. Several *in vivo* studies of different CNS pathologies demonstrated that suppression of ASTs activation and their inflammatory response resulted in reduced disease severity and improved functional recovery [73]. In addition, recent reports showed clinical improvement in patients with Alzheimer and related disorders following the perispinal administration of Etanercept, suggesting that Etanercept has the ability to penetrate into the cerebral spin fluid in the brain at a therapeutically effective concentration [74], [75]. Further *in vivo* studies should clarify whether inhibition of NF-κB signaling or TNF-α production results in protective effects, and if the NF-κB pathway results a convenient new target for the development of therapeutic strategies for the treatment of CNS commitment in HUS patients.

Given the narrow interaction between ASTs and brain ECs, local concentration of secreted astrocytic factors in response to Stx is extremely relevant to understand the role of cerebral inflammation and its relation with the microvascular injury and BBB alterations in HUS. Primary alteration of the BBB after STEC infection as a consequence of the systemic inflammation and/or bacterial-derived factors may leave the brain unprotected to the entry of Stx and LPS. Thereafter, ASTs inflammatory response creates an amplification loop that potentiates the initial endothelial damage affecting even more the integrity of the BBB. Results from this work and the bibliographical precedents, stimulate the accomplishment of a more detailed *in vivo* study to determine the contribution of brain inflammatory response, and particularly of perivascular ASTs to understand the neuropathology of HUS.

## Materials and Methods

### Ethics statement

Human normal samples were obtained from voluntary donors. This study was performed according to institutional guidelines (National Academy of Medicine, Buenos Aires, Argentina) and received the approval of the institutional ethics committee and written informed consent was provided by all the subjects.

### Human donors

Human normal blood samples were obtained from voluntary donors by venipuncture and drawn directly into plastic tubes containing 3.8% sodium citrate. Human umbilical cords were obtained from normal placentas, and placed in a sterile container filled with a transfer buffer.

### Shiga toxin-1 (Stx1) preparation

Stx1 was kindly provided Dr Sugiyama Junichi (Denka Seiken CO Ltd, Nigata, Japan). Purity was analyzed by the supplier by high performance liquid chromatography (HPLC). Stx1 preparation was checked for endotoxin contamination by the *Limulus amoebocyte* lysate assay and contained <40pg lipopolysaccharide (LPS)/µg of pure protein.

### PMN purification and platelets preparation

Human PMN were isolated by Ficoll-Hypaque gradient centrifugation (Ficoll Pharmacia, Uppsala; Hypaque, Wintthrop Products, Buenos Aires, Argentina) and dextran sedimentation, as previously described [76]. Viability was assessed by trypan blue exclusion and purity was determined by Turk's solution staining. Only fractions containing at least 80% of PMN were used.

Platelet rich plasma (PRP) was obtained by centrifugation of the blood samples (180×*g* for 10 min). For washed platelet suspensions, PRP was centrifuged in the presence of prostacyclin (PGI_2_, 75 nM), and the platelets were then washed in washing buffer (140 mM NaCl, 10 mM NaHCO_3_, 2.5 mM KCl, 0.5 mM Na_2_HPO_4_, 1 mM MgCl_2_, 22 mM sodium citrate, 0.55 mM glucose, 0.35% BSA, pH 6.5) washed platelets were resuspended in Tyrode's buffer and the platelet number was adjusted.

### ASTs isolation

ASTs were prepared from rat cerebral tissue cortex as previously described [77]. Briefly, cerebral hemispheres were dissected out from newborn rats, free of meninges, and dissociated by gentle pipetting on DMEM/Ham's F12 media (GIBCO, Invitrogen) (1∶1 v/v) containing 5 µg/ml streptomycin and 5 U/ml penicillin, supplemented with 10% fetal calf serum (FCS) (GIBCO). The cell suspensions were seeded into poly-L-lysine-coated 75 cm^2^ tissue culture flasks (Corning). After 14 days in culture, ASTs were separated from microglia and oligodendrocytes by shaking twice, for 24 h each, in an orbital shaker. The purity of ASTs cultures was 90–95%, as assessed by GFAP immunostaining by flow cytometry.

### ASTs culture and treatments

ASTs (7×10^4^) were seeded into 24 well-plates and cultured in DMEM media containing 10% FCS and supplemented with 5 µg/ml streptomycin and 5 U/ml penicillin (complete DMEM). Cultures were maintained at 37°C in a humidified 5% CO_2_ atmosphere. After 24 h, ASTs were mock-treated (control) or treated with LPS (0.5 µg/ml); Stx1 (10 ng/ml) or LPS+Stx1 (Stx1 added 18 h after LPS). Purified LPS derived from *E. coli* O111:B4 (Sigma) was used.

The inhibitor (E)-3-[4-methylphenylsulfonyl]-2-propenenitril (BAY 11-7082; Biomol) was used for suppressing NF-kB activation. BAY 11-7082 (40 µM) was added to ASTs cultures 30 min before treatment. A soluble tumor necrosis factor receptor (Etanercept; Enbrel, Wyeth Inc.) was used to block ASTs secreted TNF-α action. Etanercept (5 ng/ml) was added to ASTs cultures 10 min before treatment.

### ASTs conditioned media

Conditioned medium from confluent cultures of ASTs (CM-ASTs) grown in complete DMEM was collected every 24 h. CM-ASTs from untreated ASTs (CM-Control) or CM-ASTs treated with LPS and/or Stx1 (CM−LPS, CM−Stx1 and CM−LPS+Stx1) were collected 18 h after Stx1 treatment. The CM-ASTs were centrifuged to eliminate cells and debris and sterilized by filtration. The CM-ASTs were aliquoted and stored at −80°C until used. Before CM-ASTs were employed in experimental cultures, 7 µg/ml of Polymyxin B (Px, Sigma) and an antibody (Ab) against Stx (anti-Stx; final dilution 1∶100 Toxin Technology, Catalog Number STX1-9C9; anti-STX1 alpha subunit, mouse monoclonal antibody IgG) were added to the CM-ASTs. Proper doses of the Px and anti-Stx to block the effects of remnant traces of LPS and/or Stx1 have been previously tested and validation tests are summarized in Figure S1 [13].

### Endothelial cell culture

Human umbilical vascular endothelial cells (HUVECs) were obtained by collagenase digestion according to the method of Jaffe et al [78]. Cells were seeded until confluence on 1% gelatin-coated 25-cm^2^ tissue culture flasks and identified by their cobblestone morphology and von Willebrand factor (VWF) antibody (Immunotech) binding. Cells were grown in RPMI 1640 medium (HyClone) supplemented with 10% fetal bovine serum (FBS, Gibco), heparin (100 µg/ml), endothelial cell growth factor supplement (50 µg/ml), sodium pyruvate (2 mM), L-glutamine (2 mM), penicillin (100 U/ml) and streptomycin (100 µg/ml) (Sigma Chemical) at 37°C in a humidified 5% CO_2_ incubator. HUVECs used for experiments were between first and third passages. Cultured cells were identified as endothelial by their morphology and VWF antibody binding.

### Induction of the cerebral phenotype in HUVEC

CM-ASTs was used to induce a cerebral endothelium phenotype in HUVEC (HUVECd). HUVEC (1×10^5^ cells) were seeded into 24 well-plates and maintained in complete DMEM during 24 h. Then, HUVEC were differentiated into HUVECd by incubating them with CM-ASTs. After 24 h, HUVECd already exhibited properties of cerebral endothelium and these features lasted for up to 72 h even after removal of CM-ASTs.

### Alkaline phosphatase activity determination for endothelial cell differentiation and PMN adhesion

Endothelial Alkaline phosphatase (AlkP) was determined as previously described with some modifications [79]. Briefly, HUVEC and HUVECd were washed twice with PBS and 100 µl of 1% p-nitrophenyl phosphate disodium (p-NPP) in buffer containing 1 mM MgCl_2_ and 100 mM 2-amino-2-methyl-1-propanol (Sigma) was added to each well, and incubated at 37°C for 1 h. Then, 50 µl of stop solution (2 N NaOH) was added and optical density was measured at 405 nm in Asys UVM340 Microplate Reader (Biochrom Ltd.). Enzymatic activity, defined as dephosphorylated substrate produced in a given well during one hour at 37°C was expressed in arbitrary enzymatic units (EU) and calculated with the following equation:
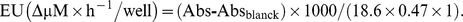



Similar procedure was applied for PMN adhesion with some modifications [80]. Briefly, purified PMN were seeded onto CM-ASTs treated HUVECd in a PMN/HUVECd ratio of 20∶1 and cultured for 3 h. Then, non-adherent PMN were removed by vigorous washing with PBS and AlkP assay was performed by incubating adherent cells for 1 h with 100 µl of 1% p-NPP in diethanolamine buffer (1 M, pH 9-8). PMN-specific AlkP activity was calculated subtracting basal AlkP activity from HUVECd alone treated with the respective CM-ASTs. Data expressed as percentages of AlkP enzymatic activity (%EA) was calculated as follow:
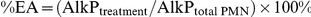
where AlkP_treatment_ was calculated as:
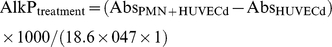
and AlkP_total PMN_ is the total AlkP from unwashed control PMN.

### ZO-1 and occludin expression

The cellular distribution of ZO-1 was evaluated microscopically on the HUVECd grown in glass coverslips. After 24 h treatment with DMEM or the different CM-ASTs, cells were pre-extracted with 0.2% Triton X-100 in buffer containing 100 mM KCI, 3 mM MgCl_2_, 1 mM CaCl_2_, 200 mM sucrose, and 10 mM Hepes (pH 7.1) for 2 min on ice and were immediately fixed with 2% paraformaldehyde in PBS (30 min on ice). The fixed cells were blocked for 30 min in 3% BSA, 0.25% Tritón X100 in PBS for 1 h. Primary antibodies (rabbit IgG anti-ZO-1, Invitrogen) were diluted in the same solution and incubated with the cells for 1 h at room temperature. Then cells were washed with PBS and secondary antibody (FITC-labeled mouse anti-rabbit (1∶200 final dilution, Vector) was incubated during 1 h and finally washed. Images were acquired using a FluoView FV1000 confocal microscope (Olympus) equipped with a Plapon 60×/1.42 objective lens and processed using FV10-ASW software (Olympus) and Adobe Photoshop CS3 using only linear adjustments.

The expression levels of ZO-1 and occludin was determined by intracellular flow cytometry (FACS) using a FACScan cytometer (Becton Dickinson) following the labeling procedure described above. For ZO-1 expression, the same antibodies were used. For occludin, mouse IgM anti-Occludin (1∶200 final dilution, Invitrogen) with PE-labeled rabbit anti-mouse IgG (1∶200 final dilution, DAKO) secondary antibody was used. Isotype matched control immunostaining was performed in parallel. FCS Express program (De Novo Sofware) for data analysis was employed. The analysis was made on at least 30,000 events per sample.

### F-actin disposition

HUVEC grown in glass coverslips were treated with DMEM or the different CM-ASTs and 24 h later cells were fixed with 1% paraformaldehyde in PBS during 20 min then washed and permeabilized with 0.25% Triton X-100. F-actin was stained using 10 µM TRITC-labeled phalloidin (Sigma) for 1 h at room temperature. F-actin distribution was evaluated by confocal microscopy using the above equipment by considering maximum fluorescence intensity projections along the whole Z-stack.

### Transendothelial Permeability Assays

HUVEC (2×10^4^ cells) were seeded onto the upper chamber of a transwell system (0.3 mm pore size, Corning) and treated with DMEM or CM-ASTs for 24 h to differentiate them into HUVECd. In some experiments, HUVECd were additionally stimulated with the different types of CM-ASTs (CM−Stx1, CM−LPS or CM− Stx1+LPS) for another 24 h. The transendothelial passage of the 44 kD glycoprotein horse radish peroxidase (HRP) (Figure 1B) and the bioactive ∼75 kD multimeric protein Stx1 (Figure 4D) were evaluated respectively as follow:

#### HRP translocation assay

The medium in the upper chamber was removed and replaced by serum-free DMEM containing 0.5 µmol/L (∼2.2 U/ml) HRP (Sigma) and in the lower chamber fresh DMEM was added. After 20 min. at 37°C the activity of HRP was evaluated in the lower chamber using commercial TMB substrate (Sigma). Briefly, 50 µl of TMB was added to 50 µl of the recovered medium in the lower chamber. After 10 min reaction was stopped using 100 µl of 1 M H_2_SO_4_ and optical density was determined at 450 nm. Relative permeability to HUVEC was calculated as follows:
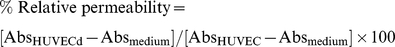



#### Stx1 translocation assay

The medium in the upper chamber was removed and replaced by serum-free DMEM containing 30 ng/ml of Stx1 while in the lower chamber fresh DMEM was added. After 30 min. at 37°C, the presence of Stx1 was determined in the lower chamber by evaluating Vero cytotoxicity [81]. Briefly, 2×10^4^ Vero cells were seeded into a 96-well plate and cultured in complete DMEM. After 24 h the medium was replaced with 0.1 ml of the supernatants collected from the lower chamber of the transwell and incubated for 24 h. Then, cells were washed with PBS and viable cells were fixed and stained with 0.2% crystal violet in 20% methanol for 20 min at 37°C. Samples were washed and lysed with 30% acetic acid solution. The resultant absorbance (Abs) was measured at 550 nm. The percentage of Vero cytotoxicity was calculated by the following formula:
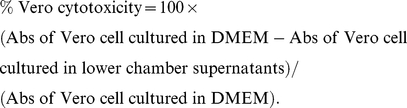



### Toxicity of CM-ASTs over HUVECd

HUVECd (1×10^5^ cells) grown in 24 well-plates were stimulated with the different CM-ASTs during 24, 48, and 72 h. After incubation, dead cells were washed away with PBS and remnant cells were fixed and stained with crystal violet. Cytotoxicity on treated HUVECd was determined microscopically using the following equation:
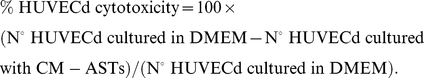



### Stx1 receptor expression

Globoside Gb_3_ (CD77) expression was evaluated on HUVECd stimulated by 24 h with the different CM-ASTs. As a positive control, cells were stimulated with 0.5 µg/ml LPS. After incubation, HUVECd were harvested, blocked with 3% BSA for 20 min and exposed to rat anti-CD77 monoclonal antibody (clone 38-13; final dilution 1∶100; Immunothech) for 45 min at 4°C. Then, cells were washed and incubated with FITC-conjugated secondary goat antibody F(ab)′_2_ anti-rat μ chain (final dilution 1∶200, Jackson ImmunoResearch Lab) in the dark for 30 min at 4°C. Immunostained cells were then washed and immediately analyzed by FACS as indicated above. Isotype matched control immunostaining was performed in parallel.

### Cell death analysis

The cell death analysis of HUVECd treated 24 h with the different CM-ASTs was performed 24 h after stimulation with a sub-toxic dose of Stx1 (2 ng/ml). Detached dead cells were carefully collected along with undetached cells. The Annexin V-FITC Apoptosis Detection Kit (Calbiochem) was used according to manufacturer's instructions. Briefly, ASTs were incubated for 30 min with media binding reagent and fluorescein isothiocyanate (FITC)-labeled annexin V (AV, 1 µg/ml) in the dark at room temperature. Cells were washed, resuspended in media binding buffer, and incubated for 10 min with propidium iodide (PI, 15 µg/ml). The percentage of total death PI^+^ AV^+/−^ (PI^+^/AV^−^ plus PI^+^/AV^+^) and PI^−^ AV^+^ cells was determined by FACS as indicated above.

### Evaluation of cell activation

To evaluate HUVECd, PMN and platelet activation, ICAM-1, E-selectin and CD11b, CD66b and P-selectin expression were determined respectively by direct immunofluorescence using conjugated anti-human monoclonal antibodies and the surface expression of these activation markers was evaluated by FACS. HUVECd were stimulated with the CM-ASTs during 12 h or 6 h at 37°C for ICAM-1 and E-selectin, respectively. Cells were washed and resuspended in PBS 3% BSA and stained with a mouse monoclonal antibody phycoerythrin (PE)-labeled anti-human CD54 (final dilution 1∶50, BD Pharmingen) for ICAM-1 determination or with a mouse monoclonal antibody fluorescein isothiocyanate (FITC)-labeled anti-human CD62E (final dilution 1∶150, Immunotech) for E-selectin determination. PMN were stimulated with the different CM-ASTs for 90 min or 40 min for CD11b or CD66b determination, respectively. Then PMN were collected in PBS 3% BSA and stained with PE-CD11b (final dilution 1∶250, Immunotech) and FITC-CD66b (final dilution 1/150, Immunotech). Platelets were stimulated with the different CM-ASTs for 1.5 h at 37°C, washed and resuspended in PBS 3% BSA and stained with a FITC-anti CD62P (anti-P-selectin antibody, final dilution 1∶50 BD Biosciences). Samples were incubated with the specific antibody for 30 min at room temperature. Then, cells were washed and suspended in 0.2 ml of ISOFLOW. In all cases, controls of isotype-matched antibody were assayed in parallel. Data were acquired and processed according the above indications. Cells were identified and gated according to their respective forward and light scattering (FSC/SSC) dot-plot profiles.

### PMN adhesion (alkaline phosphatase)

Purified PMN were seeded onto CM-ASTs treated HUVECd in a PMN/HUVECd ratio of 20∶1 and cultured for 3 h. Then, non-adherent PMN were removed by vigorous washing with PBS. Alkaline phosphatase (AlkP) assay was performed in order to measure PMN adhesion [80]. Briefly, adhered cells were incubated for 1 h with 100 µl of 1% p-NPP in diethanolamine buffer (1 M, pH 9-8) and PMN AlkP activity was determined as indicated above subtracting basal AlkP activity from HUVECd alone treated with the respective CM-ASTs. Data expressed as percentages of AlkP enzymatic activity (% EA) was calculated as follow:
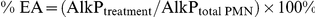
where AlkP_treatment_ was calculated as:
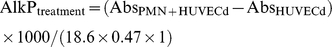
and AlkP_total PMN_ is the total AlkP from unwashed control PMN.

### PMN mediated cytotoxicity

PMN were seeded onto CM-ASTs treated HUVECd or onto a transwell coculture system to avoid contact (20∶1 PMN/HUVECd ratio). After 8 h, non-adherent PMN and detached dead HUVECd were removed by vigorous washing. Remnant cells were stained with violet crystal. To assess PMN-mediated HUVECd cytotoxicity, 3 random fields per treatment, were photographed under 600×magnifications (Nikon, Coolpix 4500 digital camera; Nikon, Eclipse TE2000-S, inverted microscope). Cell counts were performed as previously described [13] using ImageJ software (U. S. National Institutes of Health, Bethesda, Maryland). Percentage of cytotoxicity was calculated as:
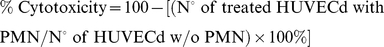



### Platelet adhesion (acid phosphatase)

Washed platelet (Pt) were seeded over treated HUVECd in a Pt/HUVECd ratio of 40∶1 and cultured for 3 h. Then, non-adherent Pts were removed by vigorous washing with warm PBS. Acid phosphatase (AcP) assay was performed in order to measure Pt adhesion [82] Briefly, adherent Pts were incubated for 1 h with 150 µl of 5 mM p-NPP in citrate buffer (0.1 M, pH 5,4) containing 0.1% Triton X-100 and enzymatic activity was determined as indicated above. Arbitrary AcP units (EU) were normalized to total EU present in unwashed control wells seeded with equal number of Pts (EU_total Pt_) according to the following equation: % EA = (EU/EU_total Pt_)×100%

### Measurement of von Willebrand factor

von Willebrand factor (vWF) release was determined by ELISA on supernatants from HUVECd after 24 h stimulation with the different CM-ASTs following the manufacturer instructions (Dako, Glostrup, Denmark). Results are expressed in ng/ml using normal pooled plasma as standard (National Institute for Biological Standars and Control, UK).

### PMN transendothelial migration

HUVECd grown in the upper chamber of transwell were treated 24 h with the different CM-ASTs and then PMN were added (20∶1 PMN/HUVECd ratio). After 1.5 h the number of PMN that migrated across the HUVECd monolayer were collected in the lower chamber of the transwell and counted microscopically using a Neubauer chamber.

### Statistical analysis

Statistical differences among treatments were determined using paired *t*-test for two groups comparison and paired one-way analysis of variance (anova) followed by Bonferroni post test for multiple comparison. A *p* value<0.05 was considered significant. All tests were carried out using Prism 5.0 (Graph Pad Software, La Jolla, CA).

### Accession numbers

TNF-α P16599 (TNFA_RAT) Reviewed, UniProtKB/Swiss-Prot

NF-kB Q63369 (NFKB1_RAT) Reviewed, UniProtKB/Swiss-Prot

Actin P68133 (ACTS_HUMAN) Reviewed, UniProtKB/Swiss-Prot

Occludin Q16625 (OCLN_HUMAN) Reviewed, UniProtKB/Swiss-Prot

ZO-1 Q07157 (ZO1_HUMAN) Reviewed, UniProtKB/Swiss-Prot

Stx1 A subunit A8B1H9 (A8B1H9_ECO57) Unreviewed, UniProtKB/TrEMBL

Stx1 B subunit C8UEK0 (C8UEK0_ECO1A) Unreviewed, UniProtKB/TrEMBL

von Willebrand factor 04275 (VWF_HUMAN) Reviewed, UniProtKB/Swiss-Prot

ICAM-1 P05362 (ICAM1_HUMAN) Reviewed, UniProtKB/Swiss-Prot

E-selectin P16581 (LYAM2_HUMAN) Reviewed, UniProtKB/Swiss-Prot

CD11b P11215 (ITAM_HUMAN) Reviewed, UniProtKB/Swiss-Prot

CD66b P31997 (CEAM8_HUMAN) Reviewed, UniProtKB/Swiss-Prot

P-selectin P16109 (LYAM3_HUMAN) Reviewed, UniProtKB/Swiss-Prot

Alkaline phosphatase P09923 (PPBI_HUMAN) Reviewed, UniProtKB/Swiss-Prot

Etanercept P20333 (TNR1B_HUMAN) Reviewed, UniProtKB/Swiss-Prot

## Supporting Information

Figure S1 **Polymyxin B and anti-Stx1 antibody effectively block the activating and cytotoxic effects of LPS and Shiga toxin 1 on cell cultures.** In order to rule out that contaminating traces of LPS and/or Stx1 present in Astrocyte conditioned media (CM-ASTs) may account for the observed functional effects over HUVECd, PMN and platelets, we systematically pre-treated CM with Polymyxin B (Px) and antibodies against Shiga toxin variant 1 and 2 (anti-Stx) before conducting the cultures (M&M). **A**. Differentiated HUVEC were cultivated in complete DMEM medium alone (HUVECd) or stimulated with 0.5 µg/ml LPS in the presence (HUVECd+LPS+Px) or absence of Px (HUVECd+LPS) during 24 h. The evaluation of the levels of Gb3 was determined by FACS. Mean fluorescent intensity (MFI) of Gb3 in HUVECd (Mean± SE). *p<0.01 HUVECd+LPS vs. HUVECd, ##p<0.01 HUVECd+LPS vs HUVECd+LPS+Px (n = 3, One way ANOVA). (Right panel) Histograms of a representative experiment. The augment of Gb3 (CD77) elicited in HUVECd after the incubation with LPS (0.5 µg/ml, Sigma), is completely abrogated by the presence of Px (7 µg/ml, Sigma). **B**. Freshly isolated human neutrophils were cultivated in complete RPMI medium alone (PMN) or stimulated with 0.5 µg/ml LPS in the presence (PMN+LPS+Px) or absence of Px (PMN +LPS) during 40 min. The evaluation of the surface levels of CD11b was determined by FACS. Mean fluorescence intensity relative to isotype control (RF) of CD11b (Mean± SE). *p<0.02 PMN+LPS vs. PMN; ##p<0.01 PMN+LPS vs PMN+LPS+Px (n = 3, One way ANOVA). The strong augment of integrin CD11b expression in the cell surface of PMN treated with above mentioned dose of LPS were completely blocked we addition of Px in culture media. **C**. Vero cells were cultivated in complete DMEM medium alone (Vero) or treated with 10 ng/ml of Stx1 in the absence (Vero+Stx1), in the presence of anti-Stx antibody (Vero+ Stx1+anti-Stx1) or with CM-ASTs treated with LPS+Stx1 with anti-Stx and Polymyxin B addition (Vero+(CM−LPS+Stx1)+Px+anti-Stx). Viability of crystal violet stained Vero cell monolayers was estimated by measuring optical density at 550 nm (DO550). *p<0.05 (Vero+Stx1 vs. Vero; #p<0.05 Vero+Stx1 vs. Vero+Stx1+anti-Stx or Vero+CM+LPS+Stx1+anti-Stx). (n = 5, One way ANOVA). The toxicity of Shiga toxin variant 1 (Stx1) over Vero cells cultures was inhibited by adding neutralizing anti-Stx antibodies (final dilution 1/100, Toxin Technology). Same results were verified in Vero cells cultures conducted in the presence of CM−LPS+Stx1 pre-treated with a combination of Px and anti-Stx. Note that LPS and Stx1 concentration employed in this control experiments are far above of the contaminating traces that may be present in the CM-ASTs.
(TIF)
