# Supplementary figures and images for: Shiga Toxin 1 Induces on Lipopolysaccharide-Treated Astrocytes the Release of Tumor Necrosis Factor-alpha that Alter Brain-Like Endothelium Integrity
Source: PLoS Pathog. 2012 Mar 29;8(3):e1002632. doi: 10.1371/journal.ppat.1002632 (PMC3315494; doi:10.1371/journal.ppat.1002632)

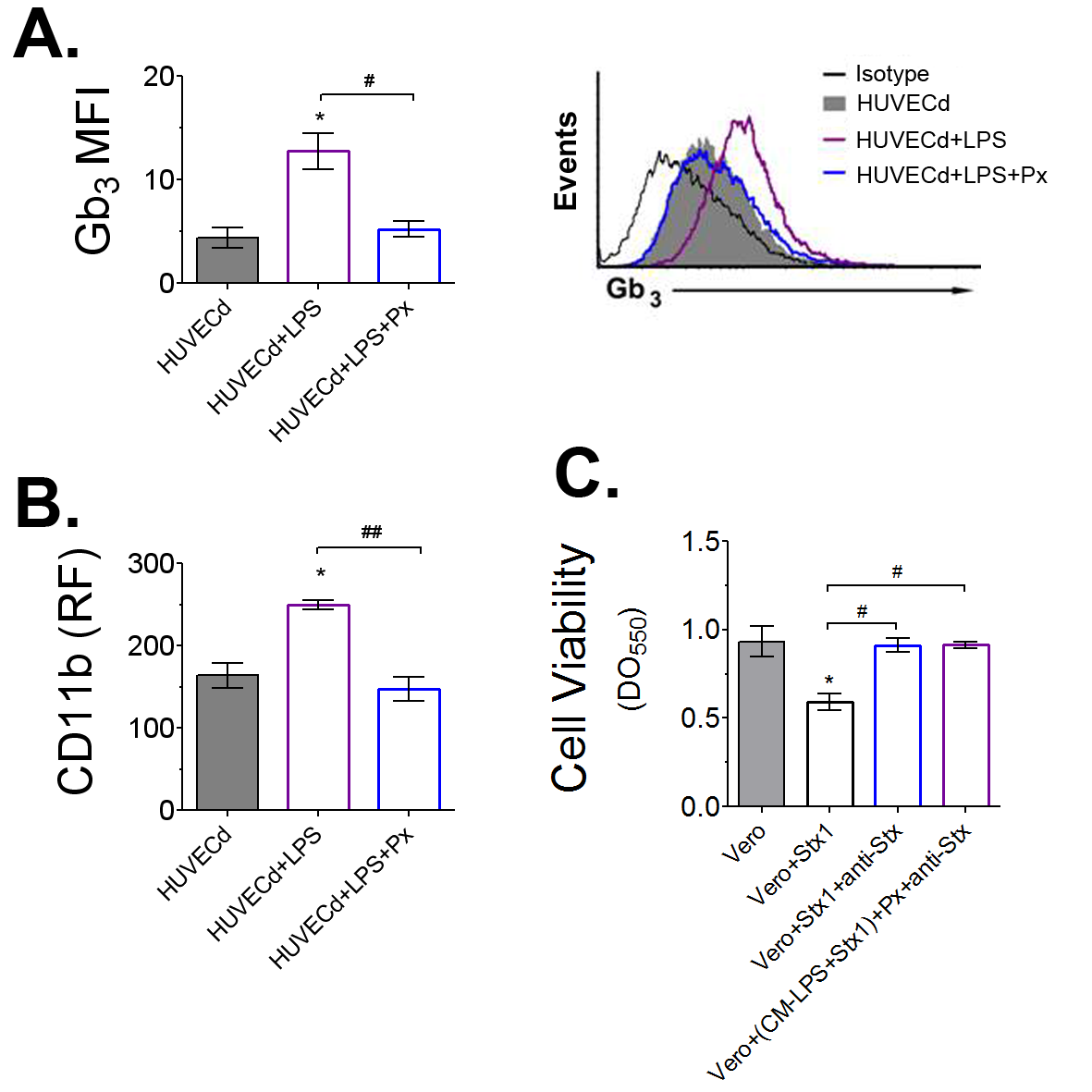

Supplement: Figure S1 — Polymyxin B and anti-Stx1 antibody effectively block the activating and cytotoxic effects of LPS and Shiga toxin 1 on cell cultures. In order to rule out that contaminating traces of LPS and/or Stx1 present in Astrocyte conditioned media (CM-ASTs) may account for the observed functional effects over HUVECd, PMN and platelets, we systematically pre-treated CM with Polymyxin B (Px) and antibodies against Shiga toxin variant 1 and 2 (anti-Stx) before conducting the cultures (M&M). A. Differentiated HUVEC were cultivated in complete DMEM medium alone (HUVECd) or stimulated with 0.5 µg/ml LPS in the presence (HUVECd+LPS+Px) or absence of Px (HUVECd+LPS) during 24 h. The evaluation of the levels of Gb3 was determined by FACS. Mean fluorescent intensity (MFI) of Gb3 in HUVECd (Mean± SE). *p<0.01 HUVECd+LPS vs. HUVECd, ##p<0.01 HUVECd+LPS vs HUVECd+LPS+Px (n = 3, One way ANOVA). (Right panel) Histograms of a representative experiment. The augment of Gb3 (CD77) elicited in HUVECd after the incubation with LPS (0.5 µg/ml, Sigma), is completely abrogated by the presence of Px (7 µg/ml, Sigma). B. Freshly isolated human neutrophils were cultivated in complete RPMI medium alone (PMN) or stimulated with 0.5 µg/ml LPS in the presence (PMN+LPS+Px) or absence of Px (PMN +LPS) during 40 min. The evaluation of the surface levels of CD11b was determined by FACS. Mean fluorescence intensity relative to isotype control (RF) of CD11b (Mean± SE). *p<0.02 PMN+LPS vs. PMN; ##p<0.01 PMN+LPS vs PMN+LPS+Px (n = 3, One way ANOVA). The strong augment of integrin CD11b expression in the cell surface of PMN treated with above mentioned dose of LPS were completely blocked we addition of Px in culture media. C. Vero cells were cultivated in complete DMEM medium alone (Vero) or treated with 10 ng/ml of Stx1 in the absence (Vero+Stx1), in the presence of anti-Stx antibody (Vero+ Stx1+anti-Stx1) or with CM-ASTs treated with LPS+Stx1 with anti-Stx and Polymyxin B addition (Vero+(CM−LPS+Stx1)+Px+anti-Stx) [file ppat.1002632.s001.tif]
